# Risk factors for avoidable hospitalizations in Canada using national linked data: A retrospective cohort study

**DOI:** 10.1371/journal.pone.0229465

**Published:** 2020-03-17

**Authors:** Lauren E. Wallar, Laura C. Rosella

**Affiliations:** 1 Dalla Lana School of Public Health, University of Toronto, Toronto, Ontario, Canada; 2 Public Health Ontario, Toronto, Ontario, Canada; 3 Institute of Clinical Evaluative Sciences, Toronto, Ontario, Canada; Osakidetza Basque Health Service, SPAIN

## Abstract

Hospitalizations for certain chronic conditions are considered avoidable for adult Canadians given effective and timely primary care management. Individual-level risk factors such as income and health behaviours are not routinely collected in most hospital databases and as a result, are largely uncharacterized for avoidable hospitalization at the national level. The aim of this study was to identify and describe demographic, socioeconomic, and health behavioural risk factors for avoidable hospitalizations in Canada using linked data. A national retrospective cohort study was conducted by pooling eight cycles of the Canadian Community Health Survey (2000/2001-2011) and linking to hospitalization records in the Discharge Abstract Database (1999/2000–2012/2013). Respondents who were younger than 18 years and older than 74 years of age, residing in Quebec, or pregnant at baseline were excluded yielding a final cohort of 389,065 individuals. The primary outcome measure was time-to index avoidable hospitalization. Sex-stratified Cox proportional hazard models were constructed to determine effect sizes adjusted for various factors and their associated 95% confidence intervals. Demographics, socioeconomic status, and health behaviours are associated with risk of avoidable hospitalizations in males and females. In fully adjusted models, health behavioural variables had the largest effect sizes including heavy smoking (Male HR 2.65 (95% CI 2.17–3.23); Female HR 3.41 (2.81–4.13)) and being underweight (Male HR 1.98 (1.14–3.43); Female HR 2.78 (1.61–4.81)). Immigrant status was protective in both sexes (Male HR 0.83 (0.69–0.98); (Female HR 0.69 (0.57–0.84)). Adjustment for behavioural and clinical variables attenuated the effect of individual-level socioeconomic status. This study identified several risk factors for time-to-avoidable hospitalizations by sex, using the largest national database of linked health survey and hospitalization records. The larger effect sizes of several modifiable risk factors highlights the importance of prevention in addressing avoidable hospitalizations in Canada.

## Introduction

Ambulatory care sensitive conditions (ACSCs) are a set of conditions for which effective and accessible preventive and primary care exists to prevent, control, or manage these conditions [1]. ACSC hospitalizations are considered avoidable with adequate primary care, and unnecessarily use health system resources [2]. In addition, ACSC hospitalizations are an indicator of health system performance. In the Canadian health system, hospitalizations for seven chronic ACSCs are routinely monitored, namely angina, asthma, congestive heart failure (CHF), chronic obstructive pulmonary disease (COPD), diabetes and diabetic complications, epilepsy, and hypertension [3]. Hospitalizations for chronic ACSCs may more specifically indicate insufficient disease management [4–9]. Studies have characterized risk factors for ACSC hospitalizations including demographics [10–19], rurality [5, 17, 20–26], socioeconomic status (SES) [1, 5, 15–17, 26–38], chronic morbidities [10, 16, 17, 39], and health system characteristics [5, 31, 32, 40–43], including access to care [16, 21, 34, 35, 44–50]. Access to care has been both positively [16, 35, 44–46] and negatively associated [34, 47–50] with ACSC hospitalizations, while other studies have found no significant association [21, 34]. However, studies of Canadian adult populations have found that increasing access to care is associated with increased risk of ACSC hospitalization, suggesting that factors outside of care must be examined to understand determinants of ACSC trends [6, 16, 35]. Certain individual-level risk factors such as income and health behaviours are not routinely collected at time of admission, and as a result, are largely uncharacterized in the context of ACSC hospitalizations [10, 16, 51, 52].

We aim to add to the evidence and address challenges encountered in previous studies. First, there is variability in ACSC definitions, ranging in the number and type of conditions included as well as age limits of study populations [1, 33, 53–55]. Second, previous studies that include income as a SES measure often utilize ecological information, which may incompletely capture the influence of SES on an individual’s risk of ACSC hospitalizations. Third, lack of a broader set of individual-level health characteristics precludes the ability to quantify the difference of potentially modifiable risk factors and broader social determinants as well as control for effects of health behaviours that are strongly linked to many ACSCs (e.g. smoking). Lastly, there are limited national cohort studies assessing risk factors for chronic ACSC hospitalizations using individual-level data that overcome challenges with interpreting cross-sectional effects [16, 19]. To address these gaps, we carry out the largest national longitudinal cohort study of risk factors for time to chronic ACSC hospitalizations using newly linked individual-level data for a nationally representative population. Specifically, this study utilizes a survival approach to identify demographic, socioeconomic, and health behavioural risk factors for chronic ACSC hospitalizations in Canada.

## Materials and methods

This study was approved by the University of Toronto Research Ethics Board, Protocol 37499.

### Data sources

#### Canadian community health survey

The Canadian Community Health Survey (CCHS) is an annual national cross-sectional survey administered conducted by Statistics Canada using computer-assisted telephone and personal interviews that collects information on sociodemographic, health status, and health care utilization [56]. The target population is Canadian citizens aged 12 years and older, representing >97% of the Canadian youth and adult population. Exclusions include those living in Aboriginal settlements, institutions (e.g. nursing homes, correctional facilities), and foster care, as well as full-time military personnel [56]. Survey participation is voluntary; however national response rates of 70–88% were achieved on survey cycles used in this study [57].

#### Discharge abstract database

The Discharge Abstract Database (DAD) is a national database of acute care inpatient separations occurring outside of Quebec, representing ~75% of such separations in Canada, maintained by the Canadian Institute of Health Information (CIHI) [58]. The DAD does not include emergency room visits. Relevant variables include admission and discharge dates, discharge disposition, diagnostic codes, and intervention codes. CIHI annually provides DAD data to Statistics Canada for statistical, linkage, and other purposes.

#### Linked CCHS-DAD

CCHS respondents were asked permission by Statistics Canada to share and link their data to other administrative records with 84.7% of respondents (excluding Quebec respondents) agreeing to share and link their data [59]. Statistics Canada probabilistically linked CCHS and DAD data using birthdate, sex, and postal code information, resulting in a cohort with baseline individual-level information and subsequent hospitalization data [59, 60]. Linked CCHS-DAD data were made available in Statistics Canada Research Data Centres in November 2017, representing the most recent linked health survey and hospital administrative data available for research use.

### Cohort creation

**Fig 1:**
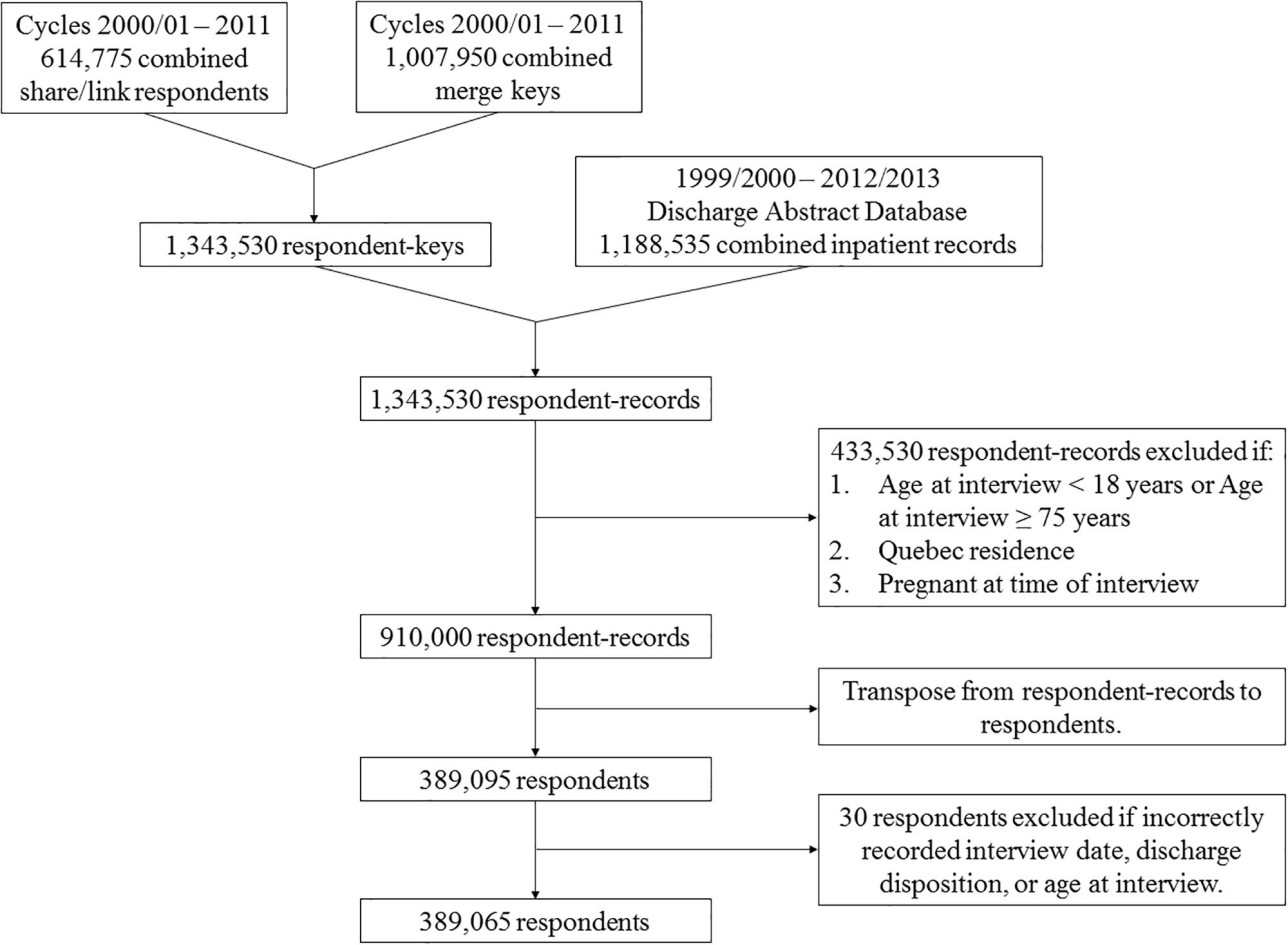
Study flow diagram depicting merger of CCHS and DAD information, transposition to person-based observations, and application of exclusion criteria to create the study cohort.

Linked CCHS and DAD data files were merged in a two-step process (Fig 1). First, eight CCHS cycles corresponding to survey years 2000/2001, 2003, 2005, and 2007–2011 were combined and merged with CCHS-DAD merge keys using household and person identification variables. This data was then merged with DAD hospitalization records from fiscal years 1999/2000–2012/2013 using the same household and person identification variables, retaining both hospitalized and non-hospitalized respondents, to create a dataset of respondent-records containing both CCHS and DAD variables. For hospitalized respondents, each row of observation represented a single hospitalization event. For non-hospitalized respondents, DAD variables were set to missing values. Respondent-records were excluded if the respondent was: 1) younger than 18 years or older than 74 years of age, 2) residing in Quebec, or 3) pregnant at time of interview. Exclusion based on older age is consistent with the CIHI definition of ACSC hospitalizations [3]. Youth were excluded to specifically identify risk factors in the adult population as pediatric ACSC hospitalization trends and risk factors are distinct from adults [28, 42]. Residents of Quebec were excluded as there are no DAD records from Quebec acute care institutions [59], and pregnant women were excluded as their behavioural risk factors may be altered from normal baseline status (e.g. change in regular alcohol intake or BMI values) [61, 62]. The merged dataset was transposed to a person-based dataset where each row of observation represented a single respondent and captured their future hospitalization status. Respondents with recording errors (e.g. an in-hospital death date prior to their CCHS interview date) were then excluded. The final study cohort consisted of 389,065 respondents, representing 63.3% of the original CCHS share/link cohort including both hospitalized and non-hospitalized respondents.

### Variable definitions

#### Dependent variable

The primary outcome is hospitalizations with a most responsible diagnosis for one of seven chronic ACSCs, namely angina, asthma, CHF, COPD, diabetes and diabetic complications, epilepsy, and hypertension, among adults aged 18–74 years old at time of admission who were discharged alive from an acute care institution. A most responsible diagnosis refers to a single diagnosis or condition attributed as the primary reason for hospitalization [63]. International Classification of Diseases -9 and -10 codes listed were used to identify hospitalizations, and intervention procedure codes were used to exclude non-avoidable hospitalizations for angina, CHF, and hypertension where certain cardiac procedures were performed at time of hospitalization [3, 16].

#### Independent variables

Variables from the CCHS were used to measure baseline information on respondent demographics (age, race, urban/rural status), SES (marital status, immigrant status, education, income), health behaviours (smoking, alcohol consumption, body mass index (BMI), physical activity), and number of chronic conditions (Alzheimer’s disease, anxiety, asthma, arthritis, back problems, bowel disease, cancer, COPD, diabetes, heart disease, high blood pressure, intestinal ulcers, migraines, mood disorders, stroke, and urinary incontinence).

### Statistical analyses

#### Descriptive statistics

Descriptive statistics were run to estimate the distribution of each independent variable in the analytic cohort, stratified by sex and type hospitalization experienced in the follow-up period. For type of future hospitalization from the baseline survey date, three mutually exclusive categories were used: avoidable, unavoidable, and none. Respondents with one or more ACSC hospitalizations in the follow-up period were categorized as avoidable. Respondents who did not experience an ACSC hospitalization but were hospitalized for one or more other conditions were categorized as unavoidable. Respondents who were either hospitalized before their interview date for any condition or were never hospitalized were categorized as none. Additional descriptive statistics were used to determine the number of respondents who experienced a ACSC hospitalization by condition, stratified by sex. Respondents were weighted using the CCHS survey weight scaled by 1/8 as eight survey cycles were combined and used in this study [64, 65]. Survey weights adjust for potential selection bias such that data is representative of the target population. Confidence intervals (CIs) were calculated using balanced repeated replication with CCHS-DAD specific bootstrap weights.

#### Regression analyses

Given the cohort study design, we applied a time-to-event survival analysis using sex-stratified Cox proportional hazard models were run to estimate the hazards associated with each independent variable and the risk of a prospective ACSC hospitalization [66]. We chose to model the data using proportional hazard models in order to directly estimate risk of hospitalization from baseline where risk factors were captured, which is appropriate given the cohort study design. Survival time was calculated as the time from the CCHS interview date to the index ACSC hospitalization event. Observations were otherwise censored at the earliest of the following events: 75^th^ birth date, discharge date with a discharge disposition of death as recorded in the DAD, or the end of the study period (March 31^st^, 2013). This method is appropriate for the cohort design and superior to other modeling approaches, such as logistic regression, which does not provide a direct estimate of risk and further the corresponding odds ratios have been shown to overestimate risk [67].

Univariable models were first minimally adjusted for age and survey cycle, to estimate the age-adjusted hazard for each independent variable on risk of ACSC hospitalization. Four models were then sequentially built, adjusted for age and survey cycle, then as follows: Model 2 (demographic variables), Model 3 (Model 2 + socioeconomic variables), Model 4 (Model 3 + behavioural variables), and Model 5 (Model 4 + number of chronic conditions). Sequential adjustment was done for model transparency and to allow for the reader to see how the effects changes before and after adjustment. Adjustment for survey cycle is a standard adjustment when combining cycles and thus was included in every model to account for potential differences across cycles over time (e.g. changes in secular population trends) given that CCHS cycles are independent cross-sectional surveys rather than a longitudinal survey of the same survey population over time [68]. Consistent with previous work, light alcohol consumption was used as the reference categorical level as this group was at lowest risk of hospitalization [69]. Confidence intervals were calculated using balanced repeated replication with CCHS-DAD specific bootstrap weights that adjusted for selection of the cohort. For fully adjusted models, the proportional hazard assumption was formally tested by evaluating the significance of interaction terms for each independent variable and survival time (α = 0.05), and visually confirmed using graphs of scaled Schoenfeld residuals.

#### Sensitivity analyses

Three sensitivity analyses were conducted to test the robustness of findings, where possible using additional available information. Specifically, we test the impact of misclassification of BMI using correction equations, misclassification of income using additional information from Statistics Canada from the Census, as well as variable follow-up time. First, the effect of potential BMI misclassification was estimated by comparing models using the original CCHS BMI variable and an adjusted BMI variable that corrects for self-reported overestimation of height and underestimation of weight using correction equations [70]. Second, the effect of household national income quintile specification was determined by comparing models with missing income modeled as a categorical level, missing income excluded, and a model using imputed income information provided by Statistics Canada through the Census. Third, a sensitivity analysis was run on a sub-cohort of respondents (CCHS cycles 2000/2001, 2003, 2005, 2007), additionally censoring observations at five years of follow-up time to produce a consistent follow-up period for all respondents to determine potential impact of variable follow-up time and minimize the impact of change in baseline risk factor status by truncating to a shorter follow-up time.

## Results

The study cohort consisted of 389,065 respondents (Fig 1). Sex-stratified baseline characteristics at time of interview for males (n = 182,335) and females (n = 206,730) are listed in Table 1. In addition, the average age for males was 43.1 ± 15.5 years and for females was 43.8 ± 14.6 years. The average survival time for males was 2,050 ± 1,235 days and for females was 2,046 ± 1,154 days. Correction for self-reported BMI values increased the proportion of obese individuals and decreased the proportion of normal and underweight individuals, particularly for underweight females.

**Table 1:** Sex-stratified baseline characteristics of pooled study participants from CCHS cycles 2000/2001-2011 (n = 389,065).

	MALES (n = 182,335)	FEMALES (n = 206,730)
Variable	Frequency (%) (95% CI)	Frequency (%) (95% CI)
**DEMOGRAPHICS**		
**Age Group**		
18–34	32.4 (32.2, 32.7)	30.6 (30.3, 30.8)
35–49	31.9 (31.5, 32.3)	32.1 (31.7, 32.6)
50–64	26.1 (25.9, 26.4)	26.7 (26.2, 27.0)
65–74	9.5 (9.4, 9.6)	10.6 (10.5, 10.8)
Missing	0.0	0.0
**Self-identified Ethnicity**		
White	78.4 (77.9, 78.9)	78.2 (77.7, 78.7)
Visible minorities^1^	21.0 (20.5, 21.5)	21.3 (20.8, 21.8)
Missing	0.6	0.5
**Urban/Rural**		
Urban	82.1 (81.8, 82.4)	83.0 (82.7, 83.2)
Rural	17.9 (17.6, 18.2)	17.0 (16.8, 17.3)
Missing	0.0	0.0
**SOCIOECONOMIC STATUS**		
**Marital Status**		
Single	26.8 (26.4, 27.2)	22.1 (21.8, 22.4)
Married or common-law	65.9 (65.4, 66.4)	64.0 (63.6, 64.4)
Separated or divorced	6.1 (5.9, 6.3)	9.6 (9.3, 9.8)
Widowed	1.0 (0.9, 1.1)	4.2 (4.1, 4.4)
Missing	0.1	0.1
**Immigrant Status**		
Canada-born	74.1 (73.6, 74.7)	73.7 (73.2, 74.2)
Immigrant	25.4 (24.9, 26.0)	25.9 (25.4, 26.4)
Missing	0.4	0.4
**Household National Income Quintile**		
Lowest	13.2 (12.9, 13.6)	17.4 (17.0, 17.7)
Lower-middle	15.1 (14.7, 15.4)	17.1 (16.8, 17.4)
Middle	17.6 (17.2, 17.9)	17.6 (17.3, 17.9)
Upper-middle	20.0 (19.7, 20.4)	17.2 (16.9, 17.5)
Highest	23.6 (23.2, 23.9)	17.4 (17.1, 17.8)
Missing	10.6	13.3
**Household Education**		
Less than secondary	5.2 (5.0, 5.4)	5.8 (5.7, 6.0)
Secondary completed	10.7 (10.4, 10.9)	10.8 (10.6, 11.0)
Some post-secondary	5.6 (5.4, 5.8)	5.9 (5.7, 6.1)
Post-secondary completed	71.9 (71.6, 72.3)	72.6 (72.2, 72.9)
Missing	6.6	4.9
**BEHAVIOURAL**		
**Smoking**^2^		
Heavy smoker	4.8 (4.7, 5.0)	2.2 (2.2, 2.3)
Light smoker	20.5 (20.2, 20.9)	17.6 (17.4, 17.9)
Former heavy	7.8 (7.6, 8.0)	4.0 (3.9, 4.2)
Former light	16.9 (16.6, 17.2)	16.2 (16.0, 16.5)
Never	46.2 (45.8, 46.7)	56.6 (56.2, 57.0)
Missing	3.7	3.2
**Alcohol Consumption**^3^		
Heavy	12.4 (12.1, 12.7)	3.9 (3.8, 4.1)
Moderate	18.1 (17.8, 18.4)	13.2 (12.9, 13.4)
Light	12.1 (11.8, 12.4)	8.5 (8.3, 8.7)
Never	46.4 (46.0, 46.9)	66.5 (66.1, 67.0)
Missing	11.0	7.9
**Uncorrected BMI**^4^		
Obese	18.5 (18.1, 18.8)	16.2 (15.9, 16.5)
Over weight	40.1 (39.7, 40.5)	26.0 (25.6, 26.4)
Normal weight	38.4 (38.0, 38.8)	50.0 (49.6, 50.4)
Under weight	1.1 (1.0, 1.2)	3.9 (3.7, 4.0)
Missing	2.0	4.0
**Corrected BMI**^4^		
Obese	24.6 (24.2, 25.0)	22.0 (21.6, 22.3)
Over weight	41.2 (40.8, 41.7)	29.1 (28.7, 29.5)
Normal weight	31.3 (30.9, 31.7)	43.1 (42.7, 43.6)
Under weight	0.9 (0.8, 1.0)	1.8 (1.7, 2.0)
Missing	2.0	4.0
**Physical Activity**^5^		
Inactive	44.6 (44.1, 45.1)	49.2 (48.7, 49.7)
Moderate	23.9 (23.6, 24.3)	25.3 (25.0, 25.7)
Active	28.4 (28.0, 28.8)	23.9 (23.5, 24.3)
Missing	3.0	1.6
**HEALTH STATUS**		
**Chronic morbidities**^6^		
One or more	50.4 (49.9, 50.8)	59.6 (59.2, 60.0)
None	49.1 (48.7, 49.6)	40.0 (39.6, 40.4)
Missing	0.5	0.3

^1^Black, Korean, Filipino, Japanese, Chinese, Aboriginal, South Asian, South East Asian, Arab, West Asian, Latin American, Other, Multiple.

^2^Heavy smoker (Currently smokes 1 or more packs per day), Light smoker (Currently smokes less than 1 pack per day), Former heavy smoker (Formerly smoked 1 or more packs per day), Former light smoker (Formerly smoked less than 1 pack per day), Never smoked (Less than 100 cigarettes smoked across the lifetime).

^3^Heavy drinker (Drinks at least once per week, and had more than 14 drinks if female or 21 drinks if male), Moderate drinker (Drinks at least once per week, and had 3–14 drinks if female and 4–21 drinks if male), Light drinker (Drinks at least once per week, and had 2 or fewer drinks if female and 3 or fewer drinks if male), Never drinker (Did not drink in the past 12 months prior to the interview date or drinks less than once per week).

^4^Obese (≥ 30 kg/m^2^), Over weight (25–29.9 kg/m^2^), Normal weight (18.5–24.9 kg/m^2^), Under weight (< 18.5 kg/m^2^).

^5^Inactive (Less than 1.5 metabolic equivalents per day), Moderate (1.5–2.9 metabolic equivalents per day), Active (3.0 or more metabolic equivalents per day).

^6^Alzheimer’s Disease or other dementia, anxiety disorder (excluding CCHS cycle 1.1 (survey year 2000/2001)), arthritis or rheumatism, asthma, back problems excluding fibromyalgia and arthritis, bowel disorders, cancer, COPD or emphysema or chronic bronchitis, diabetes, heart disease, high blood pressure, stomach or intestinal ulcers, migraines, mood disorder, stroke, urinary incontinence.

**Fig 2:**
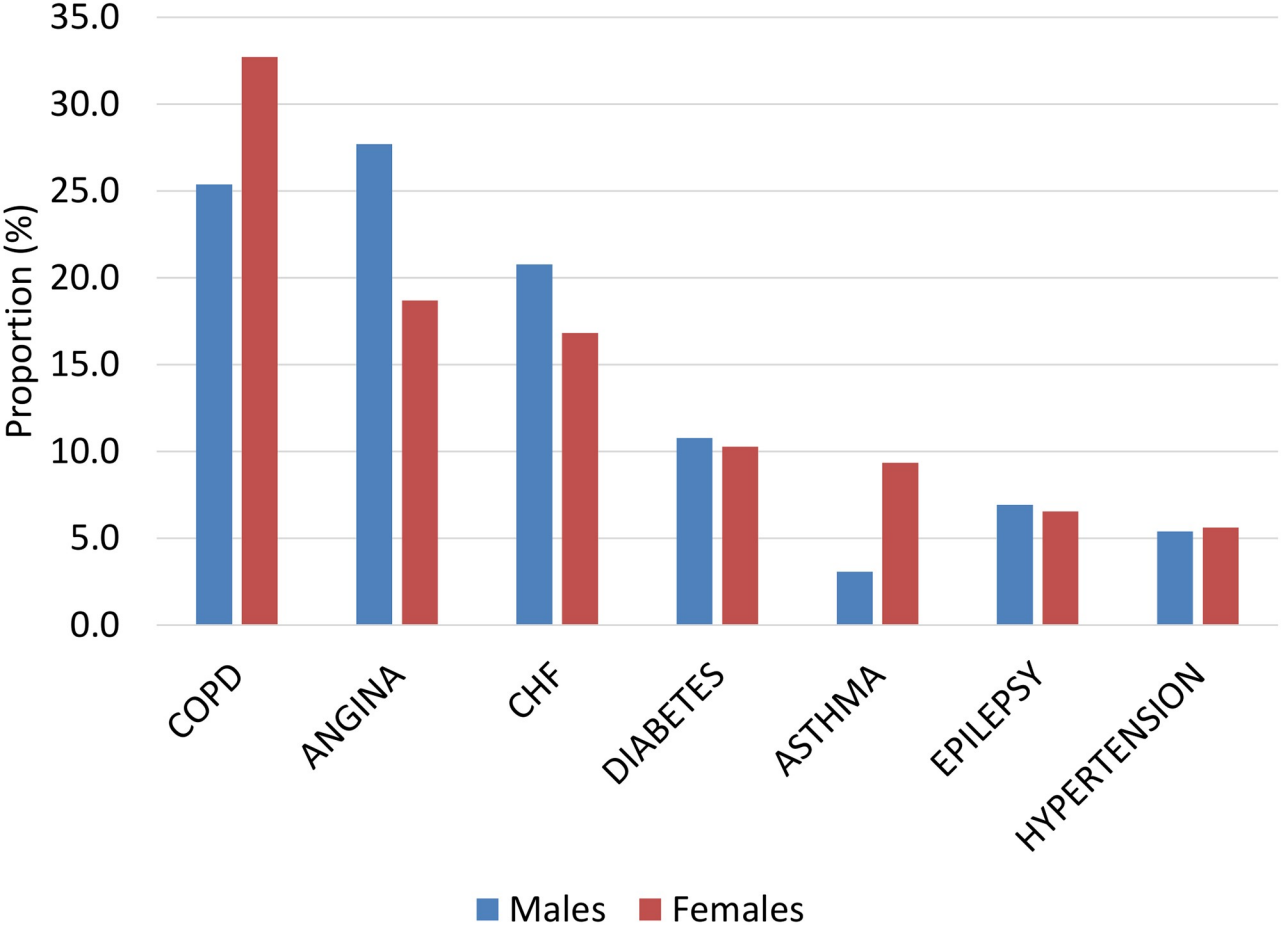
Distribution of respondents who experienced one or more prospective ACSC hospitalizations by sex and condition.

Upon additional stratification by type of hospitalization during the follow-up time, those who had an avoidable hospitalization tended to be older, rural, lower income, less educated, heavier smokers, never drinkers, obese, inactive, and have more chronic morbidities than those who had an unavoidable hospitalization or no hospitalization (Table 2). Both male and female respondents were most commonly hospitalized for angina, CHF, and COPD with asthma least common in males and hypertension least common in females (Fig 2).

**Table 2:** Sex-stratified baseline characteristics of pooled study participants from CCHS cycles 2000/2001-2011 according to type of hospitalization experienced during follow-up time (n = 389,065).

	MALES			FEMALES		
	AVOIDABLE (n = 4,330)	UNAVOIDABLE (n = 55,035)	NONE (n = 122,970)	AVOIDABLE (n = 4,155)	UNAVOIDABLE (n = 83,205)	NONE (n = 119,365)
	Frequency (%) (95% CI)	Frequency (%) (95% CI)	Frequency (%) (95% CI)	Frequency (%) (95% CI)	Frequency (%) (95% CI)	Frequency (%) (95% CI)
**Variable**						
**DEMOGRAPHICS**						
**Age Group**						
18–34	7.5 (5.9, 9.0)	16.7 (16.1, 17.2)	37.2 (36.8, 37.5)	8.1 (6.9, 9.7)	34.3 (33.8, 34.9)	29.3 (28.9, 29.7)
35–49	19.2 (17.1, 21.6)	26.5 (25.8, 27.2)	33.6 (33.2, 34.0)	18.2 (15.8, 20.6)	26.0 (25.3, 26.6)	35.1 (34.5, 35.6)
50–64	44.2 (41.9, 47.1)	36.8 (36.2, 37.5)	22.9 (22.6, 23.2)	45.5 (42.5, 48.7)	25.1 (24.6, 25.7)	27.0 (26.5, 27.5)
65–74	29.2 (26.8, 31.2)	20.0 (19.5, 20.5)	6.3 (6.2, 6.5)	28.3 (25.8, 30.6)	14.5 (14.2, 14.9)	8.6 (8.4, 8.8)
**Self-identified Ethnicity**						
White	86.7 (84.7, 89.2)	86.2 (85.5, 86.9)	76.1 (75.5, 76.7)	85.9 (82.6, 88.9)	82.6 (82.0, 83.2)	76.1 (75.4, 76.7)
Visible minorities	11.7 (10.0, 14.3)	13.2 (12.6, 14.0)	23.3 (22.7, 23.9)	13.1 (10.2, 16.5)	17.0 (16.4, 17.6)	23.4 (22.8, 24.0)
**Urban/Rural**						
Urban	74.2 (72.2, 76.1)	77.5 (76.9, 78.1)	83.5 (83.1, 83.9)	77.8 (75.3, 79.2)	80.5 (80.1, 81.0)	84.1 (83.8, 84.5)
Rural	25.8 (23.9, 27.8)	22.5 (21.9, 23.1)	16.5 (16.1, 16.9)	22.2 (20.8, 24.7)	19.4 (19.0, 19.9)	15.9 (15.5, 16.2)
**SOCIOECONOMIC STATUS**						
**Marital Status**						
Single	11.7 (10.4, 13.9)	16.2 (15.6, 16.8)	30.0 (29.5, 30.4)	10.1 (8.5, 12.0)	18.2 (17.8, 18.7)	23.9 (23.6, 24.3)
Married or common-law	73.3 (70.8, 75.5)	74.1 (73.2, 74.9)	63.6 (63.0, 64.1)	59.6 (57.0, 62.4)	66.7 (66.1, 67.3)	62.8 (62.3, 63.4)
Separated or divorced	10.8 (9.5, 12.2)	7.8 (7.4, 8.2)	5.6 (5.4, 5.8)	16.2 (14.9, 18.6)	9.3 (9.0, 9.7)	9.5 (9.2, 9.8)
Widowed	4.2 (3.1, 5.1)	1.9 (1.7, 2.1)	0.7 (0.6, 0.8)	13.1 (12.0, 15.2)	5.6 (5.4, 5.8)	3.5 (3.3, 3.7)
**Immigrant Status**						
Canada-born	78.3 (75.4, 80.9)	78.4 (77.6, 79.1)	72.9 (72.3, 73.5)	82.8 (78.9, 85.3)	78.7 (78.1, 79.4)	71.3 (70.7, 72.0)
Immigrant	20.8 (18.0, 23.4)	21.3 (20.6, 22.1)	26.6 (26.0, 27.3)	17.2 (14.1, 20.3)	21.0 (20.4, 21.6)	28.2 (27.6, 28.8)
**Household National Income Quintile**						
Lowest	22.5 (20.8, 25.1)	13.6 (13.1, 14.2)	13.0 (12.6, 13.4)	31.3 (28.8, 33.8)	18.4 (17.9, 18.9)	16.7 (16.3, 17.1)
Lower-middle	19.2 (17.5, 21.7)	16.2 (15.6, 16.9)	14.6 (14.3, 15.0)	22.2 (19.4, 25.3)	17.2 (16.7, 17.6)	17.0 (16.6, 17.4)
Middle	15.0 (13.4, 16.7)	17.8 (17.2, 18.4)	17.6 (17.2, 18.0)	12.1 (10.9, 14.3)	17.5 (17.0, 17.9)	17.7 (17.3, 18.1)
Upper-middle	14.2 (12.9, 16.5)	19.3 (18.7, 19.9)	20.3 (19.9, 20.8)	12.1 (10.4, 14.7)	17.1 (16.7, 17.6)	17.3 (16.9, 17.7)
Highest	15.0 (13.1, 16.9)	23.2 (22.6, 23.9)	23.8 (23.3, 24.2)	8.1 (7.2, 10.1)	17.1 (16.6, 17.6)	17.7 (17.3, 18.2)
**Household Education**						
Less than secondary	15.0 (13.9, 17.0)	8.3 (8.0, 8.7)	4.2 (4.0, 4.3)	20.2 (18.4, 22.3)	7.9 (7.6, 8.2)	4.7 (4.5, 4.9)
Secondary completed	13.3 (11.4, 14.9)	12.1 (11.6, 12.6)	10.3 (10.0, 10.5)	14.1 (12.0, 15.5)	11.4 (11.1, 11.8)	10.4 (10.2, 10.7)
Some post-secondary	6.7 (5.5, 7.8)	6.1 (5.7, 6.5)	5.5 (5.2, 5.7)	8.1 (6.4, 9.7)	6.2 (5.9, 6.4)	5.7 (5.5, 6.0)
Post-secondary completed	57.5 (55.2, 60.3)	68.6 (67.9, 69.3)	73.1 (72.6, 73.6)	52.5 (49.9, 55.7)	70.3 (69.7, 70.8)	73.9 (73.5, 74.3)
**BEHAVIOURAL**						
**Smoking**						
Heavy smoker	11.7 (10.4, 13.3)	6.0 (5.7, 6.3)	4.4 (4.2, 4.6)	10.1 (8.5, 11.2)	2.7 (2.5, 2.8)	1.9 (1.8, 2.1)
Light smoker	23.3 (21.4, 25.8)	18.6 (18.1, 19.3)	21.0 (20.5, 21.4)	29.3 (27.0, 32.1)	19.4 (18.9, 19.9)	16.7 (16.3, 17.0)
Former heavy	17.5 (16.1, 19.6)	12.5 (12.0, 13.0)	6.4 (6.1, 6.6)	10.1 (8.6, 11.7)	4.8 (4.6, 5.1)	3.6 (3.4, 3.8)
Former light	19.2 (17.5, 21.6)	21.6 (21.0, 22.2)	15.5 (15.2, 15.9)	18.2 (16.0, 20.4)	18.0 (17.6, 18.5)	15.4 (15.1, 15.8)
Never	23.3 (21.2, 26.0)	37.3 (36.5, 38.0)	49.1 (48.5, 49.6)	30.3 (27.2, 33.4)	51.8 (51.2, 52.4)	59.1 (58.6, 59.7)
**Alcohol Consumption**						
Heavy	7.5 (6.5, 8.8)	10.8 (10.4, 11.3)	12.9 (12.6, 13.2)	3.0 (2.5, 4.8)	3.3 (3.1, 3.5)	4.2 (4.0, 4.4)
Moderate	15.0(13.6, 17.4)	19.9 (19.3, 20.5)	17.7 (17.2, 18.1)	8.1 (6.6, 9.2)	12.9 (12.5, 13.3)	13.4 (13.0, 13.7)
Light	10.8 (9.1, 12.4)	12.0 (11.6, 12.5)	12.2 (11.8, 12.5)	5.1 (3.9, 6.4)	8.3 (7.9, 8.6)	8.6 (8.3, 8.9)
Never	59.2 (56.9, 62.0)	47.4 (46.6, 48.1)	45.9 (45.4, 46.5)	79.8 (77.8, 82.4)	69.0 (68.4, 69.6)	65.2 (64.6, 65.8)
**Uncorrected BMI**						
Obese	31.7 (29.1, 33.9)	22.6 (22.0, 23.2)	17.1 (16.7, 17.5)	31.3 (28.1, 34.0)	18.5 (18.0, 18.9)	14.9 (14.6, 15.3)
Over weight	35.8 (33.5, 38.5)	42.0 (41.2, 42.8)	39.7 (39.2, 40.2)	26.3 (23.6, 28.0)	27.4 (26.8, 27.9)	25.4 (24.9, 25.9)
Normal weight	27.5 (25.4, 30.3)	32.8 (32.0, 33.6)	40.1 (39.6, 40.6)	33.3 (30.3, 35.7)	46.8 (46.2, 47.5)	51.7 (51.1, 52.2)
Under weight	1.7 (1.1, 3.0)	0.7 (0.6, 0.8)	1.2 (1.0, 1.3)	5.1 (3.7, 7.5)	3.6 (3.3, 3.9)	3.9 (3.7, 4.2)
**Corrected BMI**						
Obese	37.5 (35.1, 40.6)	29.7 (29.0, 30.4)	23.0 (22.5, 23.4)	37.4 (34.3, 40.1)	24.9 (24.4, 25.4)	20.4 (20.0, 20.8)
Over weight	35.0 (32.6, 37.6)	41.6 (40.9, 42.5)	41.2 (40.7, 41.8)	26.3 (24.2, 28.7)	29.9 (29.3, 30.5)	28.8 (28.3, 29.3)
Normal weight	22.5 (20.4, 25.1)	26.1 (25.4, 26.8)	32.9 (32.4, 33.4)	27.3 (25.2, 30.3)	39.6 (39.1, 40.3)	44.9 (44.3, 45.5)
Under weight	1.7 (0.9, 2.2)	0.6 (0.5, 0.7)	0.9 (0.8, 1.0)	4.0 (2.3, 6.0)	1.8 (1.6, 2.0)	1.8 (1.7, 2.0)
**Physical Activity**						
Inactive	56.7 (53.8, 59.1)	46.9 (46.1, 47.8)	43.8 (43.3, 44.3)	64.6 (61.7, 67.6)	51.0 (50.3, 51.7)	48.1 (47.5, 48.7)
Moderate	20.8 (18.9, 23.3)	23.7 (23.0, 24.3)	24.1 (23.6, 24.5)	18.2 (15.7, 20.5)	25.1 (24.5, 25.6)	25.5 (25.1, 26.0)
Active	16.7 (14.9, 18.4)	25.0 (24.3, 25.8)	29.5 (29.1, 30.0)	15.2 (12.9, 16.6)	22.3 (21.7, 22.8)	24.7 (24.2, 25.2)
**HEALTH STATUS**						
**Chronic morbidities**						
One or more	84.2 (82.7, 86.3)	65.2 (64.5, 66.0)	45.8 (45.3, 46.3)	90.9 (88.6, 91.9)	65.5 (64.9, 66.1)	56.5 (56.0, 57.0)
None	15.0 (13.6, 17.2)	34.3 (33.6, 35.1)	53.7 (53.2, 54.2)	9.1 (8.0, 11.3)	34.2 (33.7, 34.8)	43.1 (42.6, 43.6)

Table 3 presents the results of five sequentially adjusted Cox proportional hazard models from minimally adjusted (Model 1) to partially (Models 2–4) to fully adjusted models (Model 5) separately for males and females. In age- and cycle-adjusted models, most variables were associated with increased risk of an ACSC hospitalization (Model 1). In males, after adjusting for household income, household educational attainment, marital status, and immigrant status, the effect of race was attenuated (Model 3). In both males and females, further adjustment for health behaviours attenuated the effects of being separated/divorced and household income (Model 4). Final adjustment for number of morbidities (Model 5) further attenuated the effect of low household income in both sexes. In females, adjusting for number of morbidities also attenuated the effect of heavy smoking as well as being obese and physically inactive.

**Table 3:** Sex-stratified multivariable sequentially adjusted cox proportional hazard models for demographic, socioeconomic, health behavioural, and number of chronic comorbidities and of index prospective ACSC hospitalization for pooled study participants from CCHS cycles 2000/2001-2011 followed from time of interview to index ACSC hospitalization, death, or end of study (March 31, 2013) (n = 318,845).

MALES					
	MODEL 1	MODEL 2	MODEL 3	MODEL 4	MODEL 5
	AGE-ADJUSTED^1^	AGE-ADJUSTED DEMOGRAPHICS^2^	MODEL 2 + SES^3^	MODEL 3 + BEHAVIOURS^4^	MODEL 4 + CHRONIC MORBIDITIES^5^
	HR (95% CI)	HR (95% CI)	HR (95% CI)	HR (95% CI)	HR (95% CI)
**DEMOGRAPHICS**					
**Self-identified Ethnicity**					
White	1.00	1.00	1.00	1.00	1.00
Visible minorities	0.74 (0.60, 0.91)	0.77 (0.63, 0.94)	0.83 (0.66, 1.06)	0.87 (0.69, 1.11)	0.90 (0.72, 1.14)
**Urban/Rural**					
Urban	1.00	1.00	1.00	1.00	1.00
Rural	1.32 (1.19, 1.46)	1.26 (1.14, 1.39)	1.18 (1.07, 1.31)	1.13 (1.01, 1.26)	1.13 (1.01, 1.26)
**SES**					
**Marital Status**					
Single	1.22 (1.02, 1.44)		0.95 (0.80, 1.13)	0.92 (0.77, 1.09)	0.90 (0.76, 1.07)
Married or common-law	1.00		1.00	1.00	1.00
Separated or divorced	1.51 (1.32, 1.74)		1.25 (1.08, 1.44)	1.12 (0.96, 1.31)	1.02 (0.86, 1.20)
Widowed	1.76 (1.35, 2.29)		1.44 (1.09, 1.89)	1.41 (1.04, 1.90)	1.45 (1.08, 1.94)
**Immigrant Status**					
Canada-born	1.00		1.00	1.00	1.00
Immigrant	0.64 (0.54, 0.75)		0.67 (0.56, 0.79)	0.77 (0.65, 0.92)	0.83 (0.69, 0.98)
**Household National Income Quintile**^6^					
Lowest	2.71 (2.27, 3.24)		2.82 (2.30, 3.46)	2.08 (1.66, 2.62)	1.58 (1.25, 2.00)
Lower-middle	1.94 (1.60, 2.35)		2.05 (1.68, 2.51)	1.68 (1.36, 2.07)	1.47 (1.19, 1.81)
Middle	1.34 (1.11, 1.61)		1.39 (1.15, 1.69)	1.21 (0.98, 1.49)	1.11 (0.90, 1.36)
Upper-middle	1.23 (1.01, 1.48)		1.28 (1.05, 1.55)	1.21 (0.98, 1.49)	1.17 (0.95, 1.44)
Highest	1.00		1.00	1.00	1.00
**Household Education**					
Less than secondary	2.06 (1.80, 2.35)		1.43 (1.24, 1.65)	1.20 (1.02, 1.41)	1.16 (0.99, 1.37)
Secondary completed	1.31 (1.12, 1.53)		1.12 (0.95, 1.31)	1.10 (0.92, 1.31)	1.14 (0.96, 1.37)
Some post-secondary	1.43 (1.18, 1.72)		1.21 (1.00, 1.46)	1.12 (0.92, 1.37)	1.11 (0.91, 1.36)
Post-secondary completed	1.00		1.00	1.00	1.00
**BEHAVIOURAL**					
**Smoking**					
Heavy smoker	3.73 (3.12, 4.47)			3.13 (2.57, 3.81)	2.65 (2.17, 3.23)
Light smoker	2.41 (2.04, 2.84)			2.20 (1.84, 2.64)	1.99 (1.66, 2.38)
Former heavy	2.01 (1.70, 2.39)			1.88 (1.57, 2.26)	1.60 (1.33, 1.92)
Former light	1.32 (1.11, 1.57)			1.36 (1.13, 1.64)	1.25 (1.04, 1.51)
Never	1.00			1.00	1.00
**Alcohol Consumption**					
Heavy	1.12 (0.88, 1.41)			0.78 (0.62, 0.99)	0.77 (0.61, 0.98)
Moderate	0.87 (0.71, 1.07)			0.85 (0.69, 1.05)	0.87 (0.71, 1.07)
Light	1.00			1.00	1.00
Never	1.66 (1.39, 1.98)			1.33 (1.11, 1.60)	1.22 (1.02, 1.47)
**Corrected BMI**					
Obese	1.69 (1.44, 1.98)			1.59 (1.33, 1.90)	1.28 (1.06, 1.54)
Over weight	0.95 (0.81, 1.10)			1.02 (0.86, 1.22)	0.96 (0.81, 1.14)
Normal weight	1.00			1.00	1.00
Under weight	2.72 (1.73, 4.30)			2.27 (1.35, 3.83)	1.98 (1.14, 3.43)
**Physical Activity**					
Inactive	1.83 (1.60, 2.10)			1.37 (1.19, 1.59)	1.28 (1.10, 1.48)
Moderate	1.30 (1.10, 1.53)			1.24 (1.04, 1.49)	1.20 (1.00, 1.43)
Active	1.00			1.00	1.00
**HEALTH STATUS**					
**Number of Chronic Morbidities**	1.49 (1.46, 1.53)				1.37 (1.33, 1.41)
FEMALES					
	MODEL 1	MODEL 2	MODEL 3	MODEL 4	MODEL 5
	AGE-ADJUSTED^1^	AGE-ADJUSTED DEMOGRAPHICS^2^	MODEL 2 + SES^3^	MODEL 3 + BEHAVIOURS^4^	MODEL 4 + CHRONIC MORBIDITIES^5^
	HR (95% CI)	HR (95% CI)	HR (95% CI)	HR (95% CI)	HR (95% CI)
**DEMOGRAPHICS**					
**Self-identified Ethnicity**					
White	1.00	1.00	1.00	1.00	1.00
Visible minorities	0.81 (0.62, 1.07)	0.84 (0.64, 1.10)	1.02 (0.78, 1.34)	1.09 (0.83, 1.44)	1.08 (0.82, 1.42)
**Urban/Rural**					
Urban	1.00	1.00	1.00	1.00	1.00
Rural	1.24 (1.11, 1.38)	1.20 (1.08, 1.33)	1.18 (1.05, 1.31)	1.12 (1.00, 1.26)	1.15 (1.03, 1.29)
**SES**					
**Marital Status**					
Single	1.27 (1.00, 1.62)		1.08 (0.85, 1.37)	1.05 (0.82, 1.35)	0.99 (0.7, 1.27)
Married or common-law	1.00		1.00	1.00	1.00
Separated or divorced	1.71 (1.48, 1.98)		1.28 (1.09, 1.51)	1.13 (0.95, 1.34)	1.01 (0.84, 1.21)
Widowed	1.70 (1.46, 1.97)		1.26 (1.08, 1.47)	1.18 (1.00, 1.39)	1.14 (0.96, 1.34)
**Immigrant Status**					
Canada-born	1.00		1.00	1.00	1.00
Immigrant	0.52 (0.42, 0.65)		0.50 (0.41, 0.61)	0.64 (0.53, 0.78)	0.69 (0.57, 0.84)
**Household National Income Quintile**^6^					
Lowest	3.51 (2.86, 4.31)		3.12 (2.50, 3.89)	2.02 (1.61, 2.53)	1.52 (1.22, 1.91)
Lower-middle	2.42 (1.89, 3.09)		2.27 (1.76, 2.93)	1.70 (1.31, 2.21)	1.53 (1.18, 1.99)
Middle	1.41 (1.12, 1.77)		1.35 (1.08, 1.70)	1.09 (0.87, 1.37)	1.04 (0.83, 1.30)
Upper-middle	1.51 (1.17, 1.94)		1.51 (1.17, 1.95)	1.35 (1.03, 1.76)	1.31 (1.00, 1.72)
Highest	1.00		1.00	1.00	1.00
**Household Education**					
Less than secondary	2.55 (2.21, 2.94)		1.75 (1.51, 2.02)	1.36 (1.18, 1.55)	1.30 (1.13, 1.48)
Secondary completed	1.40 (1.20, 1.65)		1.16 (0.99, 1.36)	1.04 (0.88, 1.22)	1.09 (0.92, 1.28)
Some post-secondary	1.77 (1.40, 2.25)		1.45 (1.15, 1.84)	1.29 (1.00, 1.67)	1.29 (1.00, 1.66)
Post-secondary completed	1.00		1.00	1.00	1.00
**BEHAVIOURAL**					
**Smoking**					
Heavy smoker	6.66 (5.51, 8.05)			4.62 (3.85, 5.55)	3.41 (2.81, 4.13)
Light smoker	3.52 (2.98, 4.16)			3.00 (2.57, 3.51)	2.66 (2.27, 3.11)
Former heavy	2.84 (2.30, 3.51)			2.32 (1.87, 2.87)	1.93 (1.55, 2.40)
Former light	1.63 (1.35, 1.96)			1.66 (1.38, 1.98)	1.52 (1.27, 1.82)
Never	1.00			1.00	1.00
**Alcohol Consumption**					
Heavy	2.67 (1.77, 4.02)			1.47 (0.97, 2.23)	1.47 (0.97, 2.22)
Moderate	0.96 (0.71, 1.31)			0.93 (0.68, 1.27)	0.94 (0.69, 1.28)
Light	1.00			1.00	1.00
Never	2.28 (1.75, 2.98)			1.84 (1.41, 2.41)	1.67 (1.27, 2.19)
**Corrected BMI**					
Obese	1.87 (1.61, 2.17)			1.47 (1.24, 1.75)	1.13 (0.95, 1.35)
Over weight	1.02 (0.89, 1.17)			0.96 (0.82, 1.11)	0.89 (0.77, 1.03)
Normal weight	1.00			1.00	1.00
Under weight	3.86 (2.30, 6.48)			2.99 (1.72, 5.18)	2.78 (1.61, 4.81)
**Physical Activity**					
Inactive	1.75 (1.50, 2.03)			1.22 (1.04, 1.42)	1.11 (0.95, 1.30)
Moderate	1.00 (0.82, 1.24)			0.90 (0.72, 1.11)	0.88 (0.72, 1.09)
Active	1.00			1.00	1.00
**HEALTH STATUS**					
**Number of Chronic Morbidities**	1.48 (1.44, 1.51)				1.35 (1.31, 1.38)

^1^Model 1 is age- and cycle-adjusted univariate analysis of independent variables.

^2^Model 2 is age- and cycle-adjusted analysis of race and rurality variables.

^3^Model 3 is age- and cycle-adjusted analysis of race, rurality, marital status, immigrant status, national household income quintiles, and highest level of household educational attainment.

^4^Model 4 is age- and cycle-adjusted analysis of race, rurality, marital status, immigrant status, national household income quintiles, highest level of household educational attainment, smoking status, alcohol consumption, BMI, and physical activity levels.

^5^Model 5 is age- and cycle-adjusted analysis of race, rurality, marital status, immigrant status, national household income quintiles, highest level of household educational attainment, smoking status, alcohol consumption, BMI, physical activity levels, and number of chronic morbidities.

^6^ Unknown income was modelled as a separate categorical level.

In the fully adjusted model (Model 5), rural residence, lowest and lower-middle income quintiles, smoking, no alcohol consumption, being underweight, and number of chronic morbidities was associated with an increased risk of an ACSC hospitalization while immigrant status was protective in both males and females. In males, widowhood, obesity, and physical inactivity were additional risk factors and heavy alcohol consumption was protective. In females, living in a low educated household was an additional risk factor. In both males and females, smoking and being underweight were the largest risk factors for ACSC hospitalizations.

Results of sensitivity analyses are listed in S1–S3 Tables. Using corrected BMI values did not appreciably alter model regression coefficients other than BMI coefficients. In males, the effect size for being obese and underweight decreased, and in females the effect size for being obese decreased and being underweight increased (S1 Table). Using imputed income information attenuated the effect size of household national income relative to the other two models, although the direction and significance was not affected (S2 Table). Restricting the observation period to 5 years (1,825 days) did not change the pattern, direction, or significance of risk and protective factors in females. In males, the effects of rural residence and obesity were attenuated (S3 Table). In addition, the effect size of heavy alcohol consumption in males was similar in both cohorts, although the 95% CI was wider in the sub-cohort.

## Discussion

This study identified sociodemographic, behavioral and clinical risk factors for time-to-chronic ACSC hospitalizations in Canada by sex, using the largest national database of linked health survey and hospitalization records. In both males and females, increased risk of ACSC hospitalization was most strongly associated with smoking and BMI, as well as low household income, after full adjustment. Sex-specific effects were found, including differential effects of widowhood, heavy alcohol consumption, obesity, and physical inactivity. In addition, effect sizes were larger for women who smoke and abstain from alcohol compared to men. Immigrant status was associated with a lower risk of ACSC hospitalization in both sexes. With the new use of linked, individual-level information on numerous risk factors, this study importantly adds to the understanding of both well and poorly characterized risk factors for chronic ACSC hospitalizations in Canada as well as overcomes limitations of previous studies that were limited by design (cross-sectional) or variables (e.g. neighbourhood-level SES compared to individually based measures).

This study distinctively focuses on chronic ACSC hospitalizations. Prior studies using international definitions often combine both acute and chronic ACSC conditions, which may mask differential risk factors [5, 10, 71]. Canadian studies of chronic ACSC hospitalizations have identified rurality, immigrant status, lower income, smoking, underweight, and comorbidities as independent risk factors [16, 19]. However, these studies were limited in scope linking one cycle of baseline information to 3–4 years of hospitalization data and not using a time-to-event analysis. Using eight health survey cycles linked to hospitalization data to, we were able to build from these studies and have further found important demographic, socioeconomic, and behavioural risk factors through the use of more recent longitudinal data and risk-based time.

One of the unique strengths of this study is the ability to link survey data to hospitalization data. Health behaviours as risk factors for ACSC hospitalizations are not well described in the literature as they are not routinely collected at time of admission [10, 16, 51, 52]. Further, previous studies of behavioural risk factors have focused on older adult populations [10, 51, 52]. In this study, smoking was the strongest risk factors in fully adjusted models for adult males and females. Importantly, this result links public health and acute care, suggesting that a focus on upstream health behaviours, the purview of public health, will decrease risk of hospitalization and subsequently reduce demand on acute care.

There were mixed effects related to alcohol consumption, particularly among males. Notably, alcohol consumption is self-reported in the CCHS and may not accurately reflect true consumption levels, which may introduce misclassification[72]. However, it is possible that alcohol consumption as measured in this survey may not be a major risk factor for chronic ACSC hospitalizations, as related results in the literature are also mixed. In a US study of Veterans Affairs or Medicare hospitalizations, severe alcohol misuse was not associated with primary ACSC hospitalizations [52]. Similar to this study, sex-specific effects for heavy alcohol consumption with a protective effect in males was observed for age to first chronic disease among CCHS respondents residing in Ontario [69]. With respect to cause-specific outcomes, heavy alcohol consumption has been shown to lower risk of myocardial infarction and stable angina while increasing risk for other cardiovascular diseases including heart failure [73, 74]. In contrast, other studies of incident heart failure did not find a significant association with heavy alcohol consumption [75, 76], and a meta-analysis of alcohol consumption and heart failure found no significant association among individuals with high to heavy alcohol consumption [77]. Likewise, heavy alcohol consumption was not associated with acute COPD exacerbations [78] or type 2 diabetes [79] but did increase risk of hypertension [80]. However, most studies do not specifically assess hospitalization outcomes which importantly differ from disease incidence outcomes. Additional research focusing on stronger measures of consumption and cause-specific outcomes is needed to better understand the association between alcohol consumption and ACSC hospitalizations.

Socioeconomic status, measured various ways, is one of more consistent studied risk factors for chronic ACSC hospitalizations [1, 10, 16, 26, 27, 35]. One of the strengths of this study is the use of individual-level rather than neighbourhood-level socioeconomic information, thus overcoming potential ecological bias. In addition, we were able to study the effect of socioeconomic status after sequentially adjusting for additional individual-level behavioural and clinical variables. Males and females in the two lowest income quintiles were at greatest risk of hospitalization in fully adjusted models, with attenuated effects for those in middle and upper-middle income quintiles. A clear gradient was not observed, perhaps reflecting different utilization patterns across SES groups.

Immigrant status was the only factor that was protective in both males and females, likely reflective of the healthy immigrant effect that is partially associated with Canada’s immigration policies, whereby Canadian immigrants have better health status compared to domestic residents [81, 82]. Those who wish to immigrate to Canada must pass an immigration medical exam and demonstrate that they are not a danger to public health, a danger to public safety, or have conditions that will put “excessive demand on health or social services” [83]. These results concur with previous evidence demonstrating protective immigration factors for ACSC hospitalizations [19] and other studies demonstrating reduced risk of premature mortality (death before the age of 75) [84].

Potential limitations of this study include the use of one-time survey data to measure baseline risk factors for ACSC hospitalizations. Given the length of follow-up time, it is possible that risk factor status may have changed from baseline over time and thus we were unable able to control for time-varying confounding. However, a sensitivity analysis with a shortened, five-year observation period generated similar results. Censoring at death was only possible for those respondents with a discharge disposition of death in the DAD, potentially resulting in incomplete censoring for those that died outside of hospital. We were limited in sample size to model ACSC hospitalizations as one endpoint, rather than ACSC-specific outcomes and therefore we cannot comment on risk factors for specific ACS conditions nor the specific relationships between the variables we studied and specific outcomes. However, we note that ACSC are used as a health system indicator as a composite outcome and thus our outcome is meaningful for health system performance. Lastly, we were unable to account for variation in health system characteristics as this information is not available in the CCHS or DAD data sources.

Several future directions were apparent from our analysis, including investigating other measures of subjective well being and variables included in later CCHS cycles (e.g. food security) and subject-specific cycles (e.g. nutrition) that were not available in all cycles. Broader definitions of ACSC hospitalizations that include acute and vaccine-preventable conditions could also be used to understand differential risk factors for these groups of conditions [85].

## Conclusions

Together, this study represents the largest survival analysis of a broad range of risk factors for chronic ACSC hospitalizations using individual-level linked data. This study confirms the effect of low socioeconomic status on increased avoidable hospitalization risk after accounting for other individual-level information. In addition, this study extends the literature on the impact of health behaviours, identifying smoking and BMI as important and modifiable risk factors among adult Canadians. This study can be informative to health system decision-makers interested in understanding the range of factors that contribute to avoidable hospitalizations in the Canadian population.

## Supporting information

S1 Table Sex-stratified multivariable fully adjusted cox proportional hazard models for demographic, socioeconomic, health behavioural, and number of chronic morbidities and of index prospective ACSC hospitalization for pooled study participants from CCHS cycles 2000/2001-2011 followed from time of interview to index acsc hospitalization, death, or end of study (march 31, 2013) using uncorrected and corrected body mass index values (n = 318,845). (PDF)

S2 Table Sex-stratified multivariable sequentially adjusted cox proportional hazard models for demographic, socioeconomic, health behavioural, and number of chronic morbidities and of index prospective acsc hospitalization for pooled study participants from CCHS cycles 2000/2001-2011 followed from time of interview to index ACSC hospitalization, death, or end of study (march 31, 2013) (n = 318,845 when modelling missing income as a categorical level) (n = 300,870 when modelling imputed income) (n = 286,865 when excluding respondents with missing income). (PDF)

S3 Table Sex-stratified multivariable fully adjusted cox proportional hazard models for demographic, socioeconomic, health behavioural, and number of chronic morbidities and of index prospective ACSC hospitalization for pooled study participants from CCHS cycles 2000/2001-2007 followed from time of interview to index acsc hospitalization, death, or five years after time of interview (1825 days) (n = 219,595). (PDF)
