# Supplementary material for: Risk factors for avoidable hospitalizations in Canada using national linked data: A retrospective cohort study
Source: PLoS One. 2020 Mar 17;15(3):e0229465. doi: 10.1371/journal.pone.0229465 (PMC7077875; doi:10.1371/journal.pone.0229465)
Supplement: S2 Table — (PDF) [file pone.0229465.s002.pdf]

Supplementary Table 2. Sex-stratified Multivariable Sequentially Adjusted Cox Proportional Hazard Models for Demographic, Socioeconomic, Health Behavioural, and Number of Chronic Morbidities and of Index Prospective ACSC Hospitalization for Pooled Study Participants from CCHS Cycles 2000/2001-2011 Followed from Time of Interview to Index ACSC Hospitalization, Death, or End of Study (March 31, 2013) (n=318,845 when modelling missing income as a categorical level) (n=300,870 when modelling imputed income) (n=286,865 when excluding respondents with missing income).

	MALES			FEMALES		
	Missing income as categorical level	Imputed income from Statistics Canada	Missing income excluded	Missing income as categorical level	Imputed income from Statistics Canada	Missing income excluded
	HR (95% CI)	HR (95% CI)	HR (95% CI)	HR (95% CI)	HR (95% CI)	HR (95% CI)
**DEMOGRAPHICS**						
**Self-identified Ethnicity**						
White	1.00	1.00	1.00	1.00	1.00	1.00
Visible minorities	0.90 (0.72, 1.14)	0.86 (0.68, 1.10)	0.86 (0.67, 1.09)	1.08 (0.82, 1.42)	1.06 (0.79, 1.41)	1.10 (0.82, 1.49)
**Urban/Rural**						
Urban	1.00	1.00	1.00	1.00	1.00	1.00
Rural	1.13 (1.01, 1.26)	1.14 (1.02, 1.28)	1.19 (1.06, 1.33)	1.15 (1.03, 1.29)	1.16 (1.03, 1.31)	1.18 (1.05, 1.34)
**SES**						
**Marital Status**						
Single	0.90 (0.76, 1.07)	0.91 (0.76, 1.09)	0.95 (0.79, 1.14)	0.99 (0.7, 1.27)	1.00 (0.77, 1.29)	0.97 (0.75, 1.27)
Married or common-law	1.00	1.00	1.00	1.00	1.00	1.00
Separated or divorced	1.02 (0.86, 1.20)	0.97 (0.82, 1.16)	1.03 (0.87, 1.21)	1.01 (0.84, 1.21)	1.03 (0.85, 1.24)	0.99 (0.82, 1.20)
Widowed	1.45 (1.08, 1.94)	1.39 (1.02, 1.90)	1.36 (0.98, 1.87)	1.14 (0.96, 1.34)	1.16 (0.98, 1.39)	1.18 (0.99, 1.41)
**Immigrant Status**						
Canada-born	1.00	1.00	1.00	1.00	1.00	1.00
Immigrant	0.83 (0.69, 0.98)	0.85 (0.71, 1.01)	0.85 (0.70, 1.03)	0.69 (0.57, 0.84)	0.70 (0.57, 0.86)	0.70 (0.57, 0.86)
**Household National Income Quintile**						
Lowest	1.58 (1.25, 2.00)	1.49 (1.19, 1.87)	1.61 (1.27, 2.03)	1.52 (1.22, 1.91)	1.36 (1.05, 1.77)	1.51 (1.20, 1.90)
Lower-middle	1.47 (1.19, 1.81)	1.45 (1.17, 1.81)	1.49 (1.21, 1.84)	1.53 (1.18, 1.99)	1.40 (1.05, 1.86)	1.53 (1.18, 1.99)
Middle	1.11 (0.90, 1.36)	1.03 (0.84, 1.26)	1.12 (0.91, 1.38)	1.04 (0.83, 1.30)	0.97 (0.75, 1.26)	1.04 (0.83, 1.30)
Upper-middle	1.17 (0.95, 1.44)	1.08 (0.86, 1.34)	1.18 (0.96, 1.45)	1.31 (1.00, 1.72)	1.17 (0.90, 1.52)	1.31 (1.00, 1.71)

Highest	1.00	1.00	1.00	1.00	1.00	1.00
**Household Education**						
Less than secondary	1.16 (0.99, 1.37)	1.17 (0.99, 1.38)	1.20 (1.01, 1.43)	1.30 (1.13, 1.48)	1.32 (1.15, 1.52)	1.30 (1.12, 1.50)
Secondary completed	1.14 (0.96, 1.37)	1.13 (0.95, 1.36)	1.09 (0.91, 1.31)	1.09 (0.92, 1.28)	1.08 (0.91, 1.29)	1.08 (0.91, 1.29)
Some post-secondary	1.11 (0.91, 1.36)	1.12 (0.91, 1.38)	1.12 (0.91, 1.38)	1.29 (1.00, 1.66)	1.29 (1.00, 1.68)	1.34 (1.02, 1.74)
Post-secondary completed	1.00	1.00	1.00	1.00	1.00	1.00
**BEHAVIOURAL**						
**Smoking**						
Heavy smoker	2.65 (2.17, 3.23)	2.68 (2.18, 3.29)	2.68 (2.18, 3.29)	3.41 (2.81, 4.13)	3.42 (2.81, 4.16)	3.41 (2.78, 4.18)
Light smoker	1.99 (1.66, 2.38)	2.04 (1.70, 2.45)	2.02 (1.67, 2.44)	2.66 (2.27, 3.11)	2.72 (2.31, 3.20)	2.71 (2.29, 3.21)
Former heavy	1.60 (1.33, 1.92)	1.69 (1.40, 2.03)	1.61 (1.33, 1.95)	1.93 (1.55, 2.40)	1.95 (1.56, 2.44)	1.88 (1.49, 2.38)
Former light	1.25 (1.04, 1.51)	1.29 (1.06, 1.56)	1.30 (1.07, 1.57)	1.52 (1.27, 1.82)	1.51 (1.25, 1.83)	1.50 (1.23, 1.83)
Never	1.00	1.00	1.00	1.00	1.00	1.00
**Alcohol Consumption**						
Heavy	0.77 (0.61, 0.98)	0.76 (0.59, 0.96)	0.77 (0.61, 0.98)	1.47 (0.97, 2.22)	1.61 (1.05, 2.47)	1.76 (1.14, 2.70)
Moderate	0.87 (0.71, 1.07)	0.83 (0.67, 1.02)	0.84 (0.68, 1.03)	0.94 (0.69, 1.28)	0.98 (0.71, 1.37)	1.06 (0.77, 1.48)
Light	1.00	1.00	1.00	1.00	1.00	1.00
Never	1.22 (1.02, 1.47)	1.21 (1.00, 1.46)	1.20 (1.00, 1.44)	1.67 (1.27, 2.19)	1.81 (1.36, 2.42)	1.88 (1.41, 2.51)
**Corrected BMI**						
Obese	1.28 (1.06, 1.54)	1.28 (1.06, 1.54)	1.28 (1.06, 1.54)	1.13 (0.95, 1.35)	1.12 (0.93, 1.34)	1.13 (0.94, 1.36)
Over weight	0.96 (0.81, 1.14)	0.95 (0.80, 1.14)	0.97 (0.81, 1.15)	0.89 (0.77, 1.03)	0.88 (0.76, 1.03)	0.89 (0.76, 1.05)
Normal weight	1.00	1.00	1.00	1.00	1.00	1.00
Under weight	1.98 (1.14, 3.43)	2.08 (1.20, 3.60)	1.88 (1.06, 3.33)	2.78 (1.61, 4.81)	2.80 (1.59, 4.91)	2.87 (1.59, 5.15)
**Physical Activity**						
Inactive	1.28 (1.10, 1.48)	1.23 (1.06, 1.43)	1.22 (1.04, 1.42)	1.11 (0.95, 1.30)	1.11 (0.95, 1.30)	1.13 (0.96, 1.33)
Moderate	1.20 (1.00, 1.43)	1.19 (0.99, 1.42)	1.10 (0.93, 1.30)	0.88 (0.72, 1.09)	0.88 (0.71, 1.10)	0.90 (0.72, 1.13)
Active	1.00	1.00	1.00	1.00	1.00	1.00
**HEALTH STATUS**						
**Number of Chronic Morbidities**	1.37 (1.33, 1.41)	1.36 (1.32, 1.40)	1.36 (1.32, 1.40)	1.35 (1.31, 1.38)	1.35 (1.31, 1.39)	1.35 (1.31, 1.38)
