# Supplementary material for: Risk factors for avoidable hospitalizations in Canada using national linked data: A retrospective cohort study
Source: PLoS One. 2020 Mar 17;15(3):e0229465. doi: 10.1371/journal.pone.0229465 (PMC7077875; doi:10.1371/journal.pone.0229465)
Supplement: S3 Table — (PDF) [file pone.0229465.s003.pdf]

Supplementary Table 3. Sex-stratified Multivariable Fully Adjusted Cox Proportional Hazard Models for Demographic, Socioeconomic, Health Behavioural, and Number of Chronic Morbidities and of Index Prospective ACSC Hospitalization for Pooled Study Participants from CCHS Cycles 2000/2001-2007 Followed from Time of Interview to Index ACSC Hospitalization, Death, or Five Years after Time of Interview (1825 days) (n=219,595).

	MALES		FEMALES	
	Sub-cohort	Full cohort	Sub-cohort	Full cohort
**DEMOGRAPHICS**	HR (95% CI)	HR (95% CI)	HR (95% CI)	HR (95% CI)
**Self-identified Ethnicity**				
White	1.00	1.00	1.00	1.00
Visible minorities	0.89 (0.63, 1.27)	0.90 (0.72, 1.14)	1.00 (0.74, 1.36)	1.08 (0.82, 1.42)
**Urban/Rural**				
Urban	1.00	1.00	1.00	1.00
Rural	1.03 (0.89, 1.19)	1.13 (1.01, 1.26)	1.18 (1.01, 1.37)	1.15 (1.03, 1.29)
**SES**				
**Marital Status**				
Single	0.86 (0.67, 1.09)	0.90 (0.76, 1.07)	0.93 (0.69, 1.26)	0.99 (0.77, 1.27)
Married or common-law	1.00	1.00	1.00	1.00
Separated or divorced	1.22 (0.96, 1.55)	1.02 (0.86, 1.20)	1.06 (0.85, 1.32)	1.01 (0.84, 1.21)
Widowed	1.38 (1.05, 1.81)	1.45 (1.08, 1.94)	1.07 (0.87, 1.32)	1.14 (0.96, 1.34)
**Immigrant Status**				
Canada-born	1.00	1.00	1.00	1.00
Immigrant	0.70 (0.55, 0.88)	0.83 (0.69, 0.98)	0.62 (0.50, 0.78)	0.69 (0.57, 0.84)
**Household National Income Quintile**				
Lowest	1.79 (1.36, 2.36)	1.58 (1.25, 2.00)	1.76 (1.32, 2.35)	1.52 (1.22, 1.91)
Lower-middle	1.65 (1.27, 2.14)	1.47 (1.19, 1.81)	1.62 (1.17, 2.24)	1.53 (1.18, 1.99)
Middle	1.17 (0.90, 1.51)	1.11 (0.90, 1.36)	1.30 (0.99, 1.71)	1.04 (0.83, 1.30)
Upper-middle	1.13 (0.87, 1.47)	1.17 (0.95, 1.44)	1.43 (1.03, 1.99)	1.31 (1.00, 1.72)
Highest	1.00	1.00	1.00	1.00
**Household Education**				

Less than secondary	1.21 (0.97, 1.51)	1.16 (0.99, 1.37)	1.22 (1.04, 1.43)	1.30 (1.13, 1.48)
Secondary completed	1.09 (0.88, 1.37)	1.14 (0.96, 1.37)	1.01 (0.82, 1.26)	1.09 (0.92, 1.28)
Some post-secondary	1.25 (0.95, 1.64)	1.11 (0.91, 1.36)	1.16 (0.87, 1.56)	1.29 (1.00, 1.66)
Post-secondary completed	1.00	1.00	1.00	1.00
**BEHAVIOURAL**				
**Smoking**				
Heavy smoker	2.20 (1.67, 2.88)	2.65 (2.17, 3.23)	3.16 (2.39, 4.17)	3.41 (2.81, 4.13)
Light smoker	1.90 (1.49, 2.42)	1.99 (1.66, 2.38)	2.44 (2.00, 2.99)	2.66 (2.27, 3.11)
Former heavy	1.40 (1.11, 1.76)	1.60 (1.33, 1.92)	1.96 (1.51, 2.54)	1.93 (1.55, 2.40)
Former light	1.42 (1.11, 1.82)	1.25 (1.04, 1.51)	1.40 (1.15, 1.69)	1.52 (1.27, 1.82)
Never	1.00	1.00	1.00	1.00
**Alcohol Consumption**				
Heavy	0.77 (0.57, 1.04)	0.77 (0.61, 0.98)	1.24 (0.73, 2.12)	1.47 (0.97, 2.22)
Moderate	0.90 (0.69, 1.17)	0.87 (0.71, 1.07)	1.02 (0.68, 1.53)	0.94 (0.69, 1.28)
Light	1.00	1.00	1.00	1.00
Never	1.33 (1.08, 1.64)	1.22 (1.02, 1.47)	1.75 (1.23, 2.49)	1.67 (1.27, 2.19)
**BMI**				
Obese	1.19 (0.93, 1.51)	1.28 (1.06, 1.54)	1.12 (0.91, 1.37)	1.13 (0.95, 1.35)
Over weight	0.95 (0.76, 1.19)	0.96 (0.81, 1.14)	0.96 (0.78, 1.16)	0.89 (0.77, 1.03)
Normal weight	1.00	1.00	1.00	1.00
Under weight	1.65 (0.76, 3.57)	1.98 (1.14, 3.43)	2.32 (1.58, 3.41)	2.78 (1.61, 4.81)
**Physical Activity**				
Inactive	1.29 (1.05, 1.59)	1.28 (1.10, 1.48)	1.23 (0.99, 1.52)	1.11 (0.95, 1.30)
Moderate	1.04 (0.84, 1.30)	1.20 (1.00, 1.43)	0.83 (0.65, 1.06)	0.88 (0.72, 1.09)
Active	1.00	1.00	1.00	1.00
**HEALTH STATUS**				
**Number of Chronic Morbidities**	1.40 (1.35, 1.46)	1.37 (1.33, 1.41)	1.36 (1.32, 1.41)	1.35 (1.31, 1.38)
